# Paediatric pre‐B acute lymphoblastic leukaemia‐derived exosomes regulate immune function in human T cells

**DOI:** 10.1111/jcmm.17482

**Published:** 2022-07-13

**Authors:** Elham Gholipour, Houman Kahroba, Nasim Soltani, Parisa Samadi, Parisa Sarvarian, Sajjad Vakili‐Samiani, Abbas Ali Hosein Pour Feizi, Mohammad Sadegh Soltani‐Zangbar, Adel Baghersalimi, Bahram Darbandi, Aliakbar Movassaghpour, Mehdi Talebi, Roza Motavalli, Amir Mehdizadeh, Abdolhassan Kazemi, Mehdi Yousefi

**Affiliations:** ^1^ Student Research Committee Tabriz University of Medical Sciences Tabriz Iran; ^2^ Hematology and Oncology Research Center Tabriz University of Medical Sciences Tabriz Iran; ^3^ Stem Cell Research Center Tabriz University of Medical Sciences Tabriz Iran; ^4^ Departments of Toxicogenomics, GROW School for Oncology and Developmental Biology Maastricht University Maastricht Netherlands; ^5^ Center for Environmental Sciences Hasselt University Hasselt Belgium; ^6^ Master of Science Neonatal Intensive Care Nursing, Faculity of Nursing and Midwifery Tabriz University of Medical Sciences Tabriz Iran; ^7^ Pediatric Disease Research Center Guilan University of Medical Sciences Rasht Iran; ^8^ Department of Molecular Medicine, Faculty of Advanced Medical Sciences Tabriz University of Medical Sciences Tabriz Iran; ^9^ Medical Philosophy and History Research Center Tabriz University of Medical Sciences Tabriz Iran; ^10^ Research Center for Integrative Medicine in Aging, Aging Research Institute Tabriz University of Medical Sciences Tabriz Iran

**Keywords:** CD8+ T cells, immunosuppression, mRNA, regulatory T cell, tumour‐derived exosome

## Abstract

Exosomes derived from solid tumour cells are involved in immune suppression, angiogenesis and metastasis; however, the role of leukaemia‐derived exosomes has less been investigated. Hence, changes in immune response‐related genes and human T cells apoptosis co‐incubated with exosomes isolated from patients' pre‐B cell acute lymphoblastic leukaemia were evaluated in this in vitro study. Vein blood sample was obtained from each newly diagnosed acute lymphoblastic leukaemia (ALL) patient prior any therapy. ALL serum exosomes were isolated by ultrafiltration and characterized using Western blotting and transmission electron microscopy. Exosomes were then co‐incubated with T lymphocytes and the gene expressions, as well as functions of human T cells were quantified by qRT‐PCR. Apoptosis and caspase‐3 and caspase‐9 protein expression were also evaluated by flowcytometry and Western blotting analysis, respectively. Exosomes isolated from ALL patients affected T lymphocytes and elevated the apoptosis. Moreover, these exosomes altered the T cells profile into regulatory type by increasing the expression of FOXP3 and Tregs‐related cytokines, including TGF‐B and IL‐10. The expression level of Th17‐related transcription factors (RoRγt) and interleukins (IL‐17 and IL‐23) decreased after this treatment. According to our findings, exosomes derived from ALL patients' sera carry immunosuppressive molecules, indicating the possible effect of exosomes as liquid biomarkers for cancer staging.

## 1 INTRODUCTION

Acute lymphoblastic leukaemia (ALL) is the most prevalence malignancy among children and despite high rate of treatment; it is one of the main causes of death in children with cancers until now.^1^ It can be classified as B‐ALL and T‐ALL types which B‐type is the most common type of cancer in children.^2^ Chromosomal and genetic abnormalities play an important role in the pathological differentiation and proliferation of lymphoid precursor cells.^1^ According to the epidemiological studies, there is also a significant link between the influence of certain factors such as pesticides, ionizing radiation or infections on the child during pregnancy and early childhood which may flows by the development of leukaemia^3^, ^4^, ^5^ which Vineis^6^ believes that children with Pre‐B ALL are probably affected by toxic environmental elements from the beginning of their prenatal period. Vogler et al.^7^ first described Pre‐B ALL in 1978, as one of the most common type of ALLs, comprising almost 25% of ALL in childhood^8^ and as ALL is a lymphocyte disorder; these patients all are inherently characterized by a dysfunction of the immune response.^9^

Cancer cells are characterized by excessive cell proliferation, tumour cell metastasis, immune cells response neutralizing and possess therapeutic resistance mechanisms.^10^, ^11^ There are several mechanisms of cell–cell communications which help to regulate the processes in cancer progression.^12^ One of the interesting nanoscale sized vesicles called ‘exosomes’ are involved in cell–cell communications. Recent studies have highlighted the secretion of exosomes from tumour cells as important contributors to debilitate the immune system‐related antitumor activity in tumour microenvironment.^13^, ^14^ Exosomes are a subtype of EVs formed by endosomal route and are secreted by normal or malignant cells. Exosomes also contain molecules including DNA, mRNA, proteins and lipids,^15^, ^16^ and are generally identified based on their protein content (e.g. HSP70, CD63 and β‐Actin) and size (≈30–100 nm in diameter).

Cancer cells secrete large quantities of tumour cells‐derived exosomes (TEXs), which are found in all body fluids. However, the exosomes are isolated from peripheral blood samples in the most human studies.^17^, ^18^ TEXs are able to suppress immune cell functions^19^, ^20^, ^21^ and can also induce the activation as well as the expansion of human regulatory T cells (Treg) ex vivo and in vivo.^22^, ^23^ According to the recent achievements about the key role of exosome in regulation of the host immune system in cancer progression, studies have focused on TEXs and their effect on immune cells.

Suppressed anti‐tumour immunity response in patients with cancer, particularly those with advanced stage of disease leading to cancer progression.^24^, ^25^ The ability of the tumour cells to escape from the host immune system has long been considered a complication for cancer immunotherapy.^26^ TEXs carry a range of soluble factors and membrane‐bound from tumour cells, which most of them mediate immune suppression and mechanisms used by cancer cells to disrupt anti‐tumour response of immune cells,^27^, ^28^ depending upon the type of molecular cargoes carried by exosomes toward target cells. For instance, it has been reported, TEXs can inhibit the function of human CD8+ T cells via inducing apoptosis through the Fas/FasL pathway.^20^, ^29^ The protection of immune cells from TEXs‐induced dysfunction, apoptosis and suppressive signalling are probable to become considerable aspects of future therapeutic anti‐tumour strategies.^30^ Thus, improving our understanding of TEXs‐mediated cellular and molecular mechanisms for immune suppression is necessary.

Several studies demonstrated the key role of exosomes in haematological malignancies.^31^, ^32^ Recent evidence supports the role of leukaemia‐derived exosomes (LEXs) in cancer growth, as well as the treatment of human haematological diseases. Leukaemia‐derived exosomes interacting with B and T cells, monocytes, natural killer (NK) cells and granulocytes create an immunoinhibitory environment to help the escape of leukaemia cells from the immune responses.^33^ In a study, exosomes derived from sera of acute myeloid leukaemia (AML) patients have a detrimental effect on the ability of NK cells to eliminate tumour cells.^34^ Similarly, exosomes derived from multiple myeloma patients mediated the suppression of T cells via both promoting the proliferation of Treg cells and reducing the viability of CD4+ T cells.^35^ Accordingly, in view of the emerging importance of exosomes in leukaemia, the purpose of the present study was to evaluate the regulatory properties of exosomes on immune T cells profile.

## 2 MATERIALS AND METHODS

### 2.1 Study design

Vein blood samples were collected from 13 patients with acute lymphoblastic leukaemia and 13 healthy donors (as T cell‐donors). The entry and exit criteria for the patients are as follow: entry criteria: Only patients with acute lymphoblastic leukaemia (ALL) were selected for the study. The age range of patients should be between 2 and 12 years. Patients should have no history of other leukaemia or any kind of recurrent cancers. Patients' samples should be taken before starting chemotherapy and other ALL routine medications such as dexamethasone. The sample volume for ALL patients should be 3 ml. Exclusion criteria were as follows: Children with ALL but with a history of interfering underlying diseases were excluded from the study. Patients with other types of leukaemia, such as acute myeloblastic leukaemia, were excluded from the study. Patients receiving chemotherapy or other interfering drugs such as dexamethasone were excluded from the study. This study was approved by Ethics Committee at Tabriz University of Medical Sciences, Tabriz, Iran (Code: IR.TBZMED.REC.1398.1063). Written informed consent was obtained from all subjects after receiving an explanation of the study.

### 2.2 diagnostic criteria

Patients were determined based on clinical, morphological, immunological and cytogenetic characteristics. Also, the factors including the following: Age, sex, primary white blood cell count, subgroup based on classification FAB and immunophenotype affects the prognosis in children with ALL. The characteristics of the B‐cell precursor ALL patients (*n* = 13) are listed in Table S1.

### 2.3 Peripheral blood T‐lymphocyte subsets isolation

Vein blood samples (10 ml) were retrieved from 13 healthy donors and the isolation of peripheral blood mononuclear cells (PBMCs) were started by density gradient centrifugation (450 g, 20 min) (Ficoll Lymphodex, Inno‐Train, Germany) and then, obtained cells were washed two times using Roswell Park Memorial Institute (RPMI) 1640 (Sigma‐Aldrich, Chemie, Steinheim, Germany). Thereafter, total T cells were isolated by immunoaffinity‐based capture procedure using magnetic cell sorting (MACS) negative selection according to the manufacturer's instructions (Miltenyi Biotec, San Diego). In order to the isolation of T cells via MACS procedure, the anti‐biotin microbeads and biotin‐antibody cocktail were employed to isolate CD3+ T cells from the other immune cells. To ensure the purity of T cell populations they were characterized by flow cytometry (Figure 2, Right above).

The subsets of isolated T cells were washed with phosphate buffered saline (PBS) (Sigma, Germany), and cultured in RPMI 1640 containing 10% foetal bovine serum (FBS), 100 U/ml of penicillin, and 200 mM L‐glutamine at 37°C in a 5% CO_2_ humidified atmosphere and were used for experiments.

### 2.4 Exosome isolation

Vein blood samples (3 ml) were obtained from 13 newly diagnosed ALL patients without any virus infection, as a source of leukaemic cell‐derived exosome (LEX) and the serum sample was then separated from whole blood. Exosome isolation was started according to the isolation method^36^ through the serum centrifugation at 500 *g* for 30 min at 4°C to remove cellular debris (Eppendorf Refrigerated Centrifuge 5417 R). Then, the supernatant was placed into polyallomer centrifuge tubes and ultra‐centrifuged at 110,000 *g* for 2 h to remove larger extracellular vesicles, including dead cells and microbodies. Finally, 95% of the supernatant was collected and filtered using 0.22‐μm Nylon Syringe Filter. Subsequently, the samples were ultra‐centrifuged for an additional 2 h at 110,000 g (both at 4°C) for exosome depletion by TLA 100.2 rotor (Beckman coulter's ultracentrifuge, USA). Then, the exosomes were filtered by 0.22‐μm membrane filter and resuspended in PBS and increased up to 1 ml. Finally, the amount of exosomal total protein content was measured using Bicinchoninic Acid (BCA)‐assay (Cat No: DB9684‐50 ml, DNA biotech Co. Tehran, I.R. Iran) according to Walker et al.^37^ protocol. The suspension was stored at −80°C.

### 2.5 Western blotting

The Western blotting experiment was used for the characterization of isolated exosomes by the detection of exosomal surface markers including CD9, CD63, CD81 and CD19 (as B‐lymphocytes pan marker), and desired caspase‐3 and ‐9 expression levels. Briefly, after isolated exosomes lysis and cultured T lymphocytes by RIPA buffer (Santa Cruz, USA), sample buffer containing β‐mercaptoethanol added to 20–30 μg of exosomal total protein and heated at 95°C for 5 min. Then, we have determined the protein concentration of lysate using the Bradford assay (Protein Assay Kit, Razibiotech, Iran). The proteins electrophoresis was performed on 10% SDS‐PAGE and transferred to polyvinylidene fluoride (PVDF) membranes. The membranes were blocked in PBS containing 0.5% Tween‐20 plus 5% non‐fat skim milk and then separately incubated overnight at 4°C with primary antibodies (Santa Cruz, USA) according to the supplier recommended dilutions (1: 500 dilution of anti‐CD81, anti‐CD63 and anti‐CD9 for exosome lysates and anti‐Caspase‐3&9 for T‐cell lysates that already prepared). After subsequent washing, the membranes were further incubated with a conjugated secondary antibody (HRP‐conjugated antibody) solution for 1 h at room temperature and washed extensively. Finally, protein bands were detected by an ECL Western blotting substrate (Amersham ECL Select GE healthcare life sciences, USA).

### 2.6 Transmission electron microscopy (TEM)

Typical size and morphology of exosomes were evaluated by electron microscopy. To this aim, 20 μl of exosomes was loaded on a 300‐mesh copper grid and subsequently stained with ~2 drops of the uranyl acetate solution 1.5 wt/v% (TAAB, England) for 2 min at room temperature. Then, to avoid the degradation by electron beams, the grids were coated with carbon film and the excess staining solution was removed and air‐dried at room temperature for downstream imaging analysis with TEM using LEO 906 Zeiss instrument (Freiburg, Switzerland) with an accelerating voltage of 80 kV.

### 2.7 Cell culture with exosomes

We co‐incubated isolated immune T cells from healthy donors (HD) with ALL patients‐derived exosomes (100 μg/10^6^ cells)^38^ in RPMI‐1640 medium supplemented with 10% exosome‐depleted FBS (previously centrifuged for 90 min at 59,000 g followed by a 0.22‐μm filtration) and 1% penicillin–streptomycin for 24 h by providing a humidified atmosphere containing 5% CO_2_ at 37°C.^39^, ^40^ Then, cell apoptosis and certain protein expression levels were evaluated. Moreover, T cells treated with sterile PBS were considered as untreated controls.

### 2.8 Fluorescent labelling and internalization of exosomes

Fluorescent labelling of isolated exosomes was achieved using a PKH26 Fluorescent Cell Linker Kit (Sigma‐Aldrich) according to the manufacturer's protocol with some modifications.^41^ Briefly, exosomes (320 μl) were suspended in 250 μl of diluent C. Then, 1 μl of PKH26 was added to the suspension of exosomes and was incubated for 5 h at 37°C. Then, the labelled exosomes were separated from the unbound dye using ultra‐centrifuge at 59,000 g for 90 min at 4°C. Then, labelled exosome was added to HD T cells in culture media. After 4 h of incubation at 37°C, the cells were washed with PBS and analysed immediately by a flowcytometry instrument. The cells were assessed using FACS Calibur flow cytometer (BD Biosciences), and the data were analysed using FlowJo software (Becton Dickinson, Mountain View, CA).

### 2.9 RNA analysis and qRT‐PCR

Total RNA was extracted using FavorPrep™ Total RNA Kit (Favorgen, Taiwan) according to the manufacturer's protocol. Then, the quantity and quality of mRNA were confirmed by Nanodrop (ODs = 260 m) and gel electrophoresis (1.5%), respectively. At the reverse transcription step, complementary DNA (cDNA) was synthesized using a first strand cDNA synthesis BioFact™ RT‐Kit (BioFACT, Daejeon, Korea) and analysed by polymerase chain reaction (PCR). We quantified the mRNA expression by quantitative PCR (qPCR) using the 2^−ΔΔC*T*^ relative quantitation method and normalized by the expression level of GAPDH as the internal control. The primers are listed in Table 1.

**TABLE 1:** The primer sequences used in qRT‐PCR experiments

Gene	Sequence
Human Gapdh	Reverse: GCCATCACGCCACAGTTTC Forward: ACAACTTTGGTATCGTGGAAGG
Bax	Reverse: CAGCCCATGATGGTTCTGAT Forward: TTCTGACGGCAACTTCAACT
BCL‐2	Reverse: GGCAACGATCCCATCAATCT Forward: GGGAATCGATCTGGAAATCCTC
Human IL‐10	Reverse: TGGAGCTTATTAAAGGCATTC Forward: CATCGATTTCTTCCCTGTGAATCT
Human IL‐17	Reverse: GGATATCTCTCAGGGTCCTCATT Forward: CATAACCGGAATACCAATACCAAT
Human IL‐23	Reverse: CACAGGGCTATCAGGGAGC Forward: GGACAACAG TCAGTTCTGCTT
Human TGF‐beta	Reverse: GAGAGCAACACGGGT TCA Forward: CGACTACTACGCCAAGGA
FOX‐P3	Reverse: GTGGAAACCTCACTTCTTGGTC Forward: TCATCCGCTGGGCCATCCTG
RORγT	Reverse: AGTGGGAGAAGTCAAAGATGGA Forward: ACTCAAAGCAGGAGCAATGGAA

Abbreviations: F, forward primer; qRT‐PCR, quantitative real‐time polymerase chain reaction; R, reverse primer.

### 2.10 Flow cytometry

Treated cell with exosomes, were harvested after 24 h incubation and washed twice with PBS. Then, cells were resuspended in binding buffer at a concentration of 1 × 10^6^ cells/ml according to the manufacturer's instruction (BD Biosciences, USA). Subsequently, 5 μl of Annexin V‐FITC and 5 μl of propidium iodide (PI) solution were added and incubated for 30 min at room temperature in a dark room. Then, 400 μl of binding buffer was added, and the labelled cells were counted using a FACS Calibur (BD Biosciences, USA) flow cytometer. All apoptotic cells at the early stage (Annexin V‐FITC–positive, PI–negative), necrotic/late apoptotic cells (double positive), as well as living cells (double negative) were counted by flowcytometer and subsequently analysed by Cell Quest software (BectonDickinson). Untreated cells were used as the negative control.

### 2.11 ELISA

The exosomes‐treated cells were harvested after 24 h and washed with PBS at 350 *g* for 10 min at room temperature. The supernatants from cells were collected and assayed for the concentration of specific cytokines (human IL‐23, IL‐17, IL‐10 and TGF‐β) using appropriate enzyme‐linked immunosorbent assay (ELISA) kits (MyBioSource, San Diego, CA, USA) according to the manufacturer's protocol. OD was measured at 450 nm by the ELISA plate reader (BP‐800, Biohit, Finland).

### 2.12 Statistical analysis

The results were analysed by GraphPad Prism 6.0 software (GraphPad Software, Inc.) and SPSS 20.0 software (SPSS Inc). Data are expressed as mean ± standard error of mean. Data of Western blot were quantified using Image J software. A *p*‐value less than 0.05 (*p* < 0.05) indicates a statistically significant difference. Non‐parametric Wilcoxon matched‐pairs signed rank test was used to evaluate the statistical significant between the treated and untreated group.

## 3 RESULTS

### 3.1 Leukaemia‐ derived exosome characterization

**FIGURE 1:**
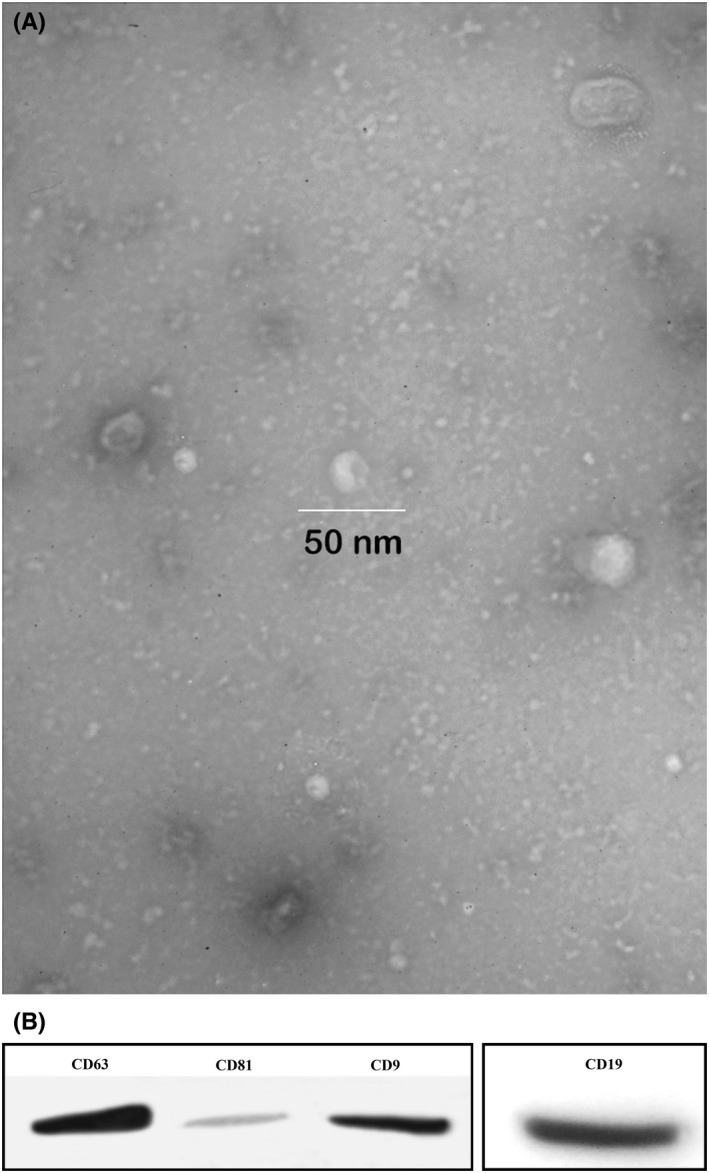
Exosome characterization. (A) Exosomes observed by TEM images of exosomes isolated from sera of patients with ALL and showed the spherical morphology, cup‐shaped morphology of MVs with the ranging in size in 50 nm with. (B) Western blotting analysis of CD9, CD63, CD81 and CD19 (as B‐lymphocytes pan marker). ALL, acute lymphoblastic leukaemia; MV, microvesicle; TEM, transmission electron microscopy

The exosomes were extracted from pre‐B ALL children via ultracentrifugation and yielded a protein concentration of 270 mg/ml as determined by BCA assay. The shape and size of the purified exosomes were evaluated by electron microscopy and showed that the exosomes had dimpled and cup‐shaped morphology of lipid bilayer‐enclosed microvesicles (Figure 1A). Western blot analysis showed the expression of exosome specific markers, including CD9, CD63, CD81 and CD19 (as B‐lymphocytes pan marker) (Figure 1B). All these data together confirmed the identity of exosomes.

### 3.2 Leukaemia‐ derived exosome internalization

**FIGURE 2:**
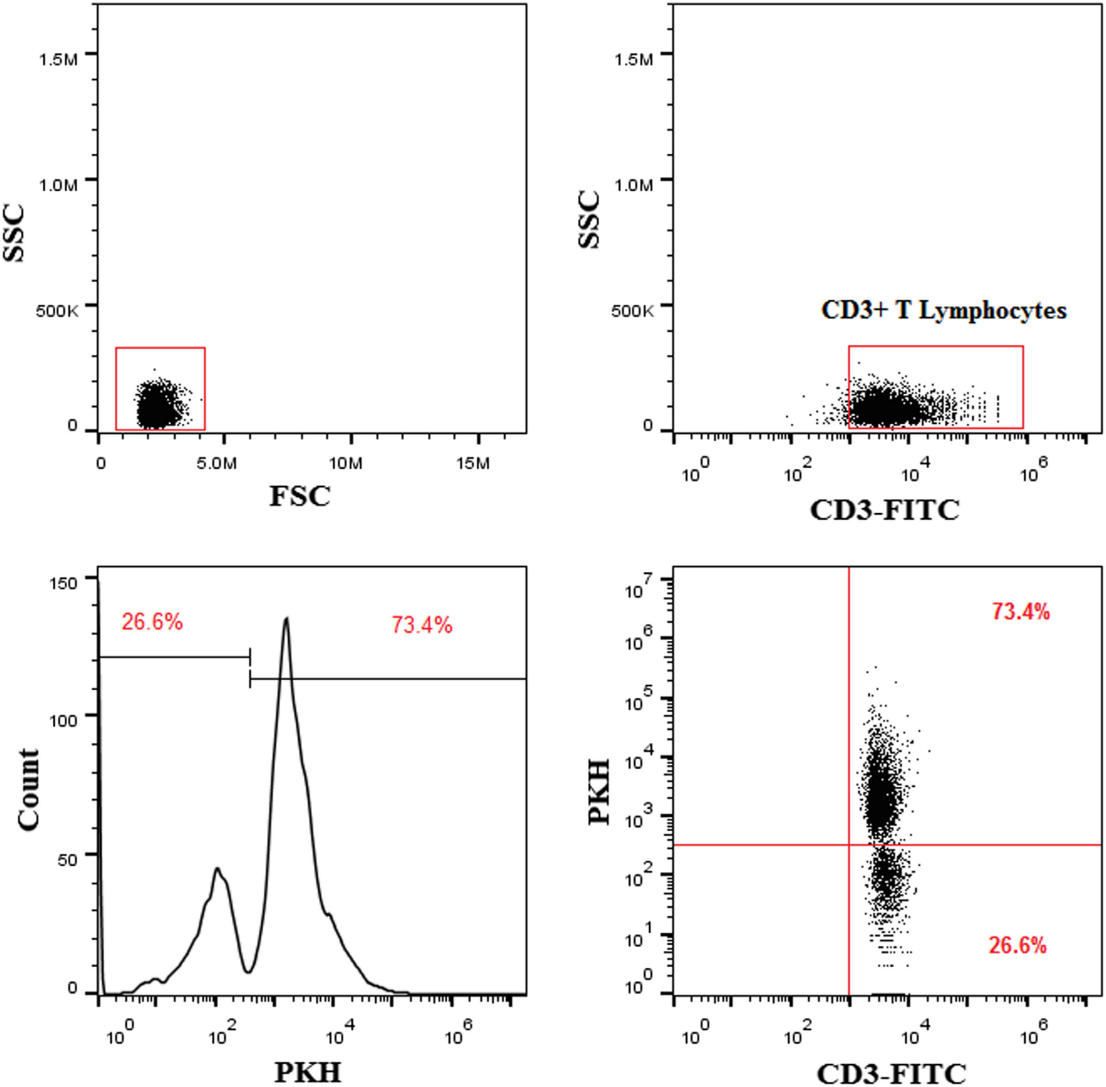
Uptake of PKH26‐labelled LEXs by human T cells. LEX was labelled with the PKH26 dye as described in methods and co‐incubated for 5 h with freshly isolated human T cells. The images are showing flow cytometry results after staining and note that most of T cells (72.35 ± 8.43) internalize exosomes. LEX, leukaemia derived exosome

LEXs were stained by Immunofluorescence staining of PKH26 to confirm the exosome uptake by T cells after incubation of PKH26‐labelled LEXs with the T cells from healthy donors (HD). The percentage of internalized exosomes (72.35 ± 8.43) was evaluated by flowcytometry (Figure 2). This observation revealed internalized LEXs by HD T cells and started subsequent signalling in recipient cells, changing gene expression profile and the role of human T lymphocytes.

### 3.3 Leukaemia‐ derived exosome ‐induced apoptosis of activated T‐cells

**FIGURE 3:**
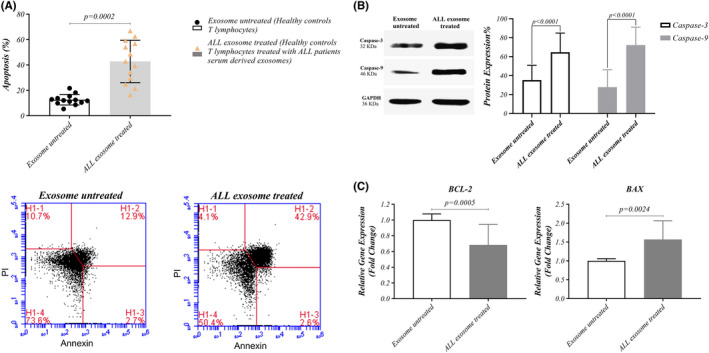
Exosomes‐mediated apoptosis in T cells treated with exosomes. (A) Representative flowcytometry data for Annexin V binding in T cells from HDs co‐incubated with exosomes isolated from patients with ALL. Exosomes of patients with ALL induced significantly more apoptosis in T cells than untreated T cells (B) Gene expression of BAX in T cells treated with exosomes is up‐regulated but BCL2 gene expression is down‐regulated rather than untreated T cells (C) Western blotting data of caspase‐3 and caspase‐9 in T cells co‐incubated with exosomes significantly increased than untreated T cells. Wilcoxon matched‐pairs signed rank test was used to evaluate the statistical significant between the treated and untreated group (*n* = 13 per group). ALL, acute lymphoblastic leukaemia; FITC, Fluorescein isothiocyanate; HD, healthy donor; UL, upper left; UR, upper right; LL, lower left; LR, lower right; PI, Propidium iodide

Our data presented that activated human T cells co‐incubated with LEXs bind to Annexin V and undergo apoptosis (Figure 3A). It has been previously reported that tumour‐derived exosomes inhibit proliferation and increase apoptosis rate.^42^, ^43^ To examine this phenomenon, the isolated exosomes from sera of ALL patients were co‐incubated with T cells for 24 h. LEXs induced significant T‐cell early apoptosis compared with the untreated controls, as evidenced by Annexin binding (42.82 ± 16.70 versus 12.62 ± 4.143, *p* = 0.0002). Additionally, the results revealed the expression of caspase‐3 (64.692 ± 20.287 versus 35.308 ± 15.628, *p* < 0.0001), and caspase‐9 (72.231 ± 18.983 versus 27.769 ± 18.435, *p* < 0.0001) as apoptotic markers after co‐incubation of HD T‐cells with LEXs (Figure 3B). In addition, Bax gene expression (1.567 ± 0.4974 versus 1.000 ± 0.05745, *p* = 0.0024) was significantly increased, while Bcl2 gene expression (0.6838 ± 0.2597 versus 1.000 ± 0.07692, *p =* 0.0005) were dramatically decreased compared with the untreated T cells (Figure 3C).

### 3.4 Effect of Leukaemia‐ derived exosomes on T cell transcription factor gene expression

**FIGURE 4:**
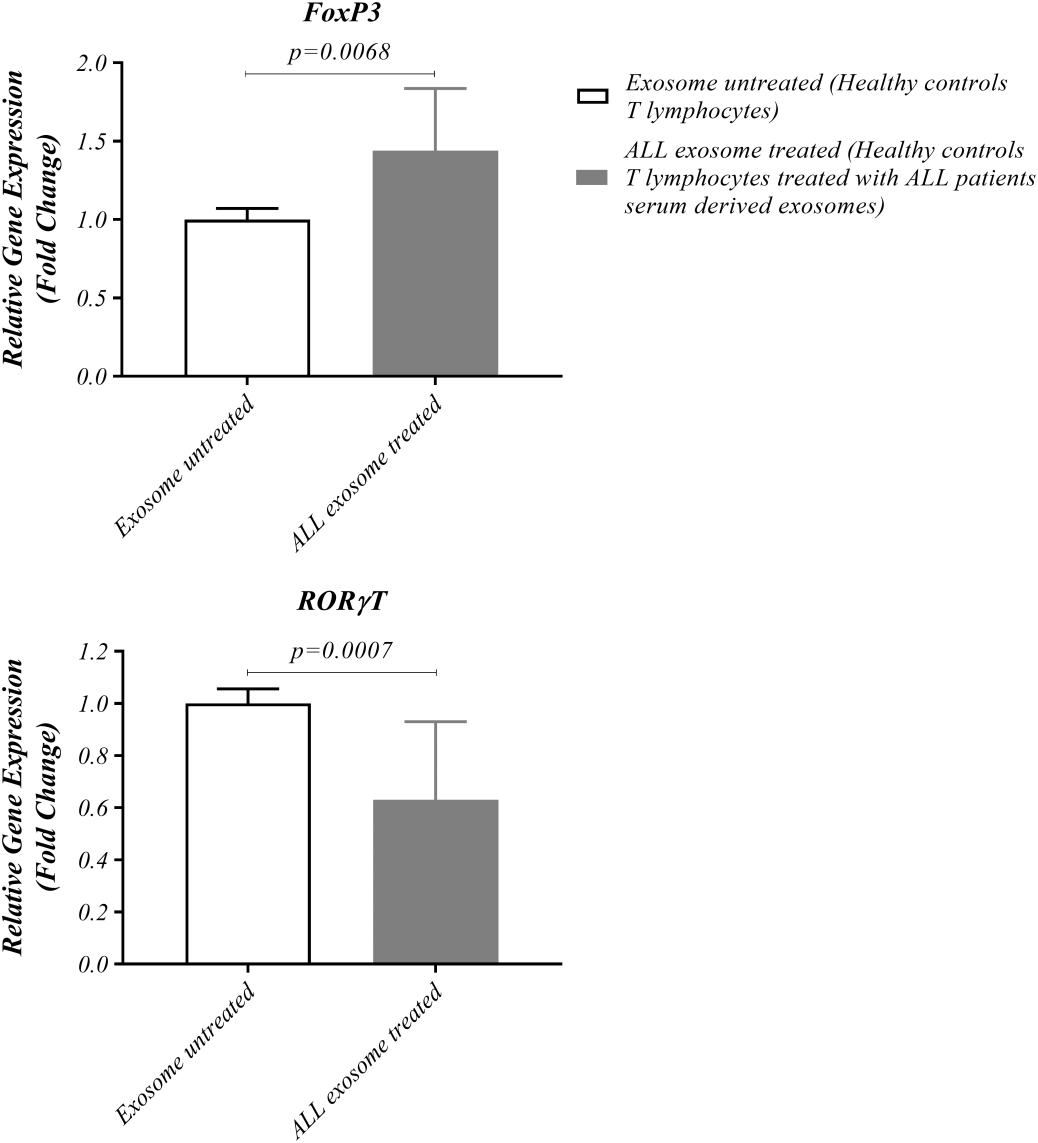
Inhibition of T‐cell proliferation by ALL cells –derived exosomes. (A) T cells from HDs co‐incubated with exosomes isolated from patients with ALL and gene expression of Foxp3 in T cells treated with exosomes is up‐regulated but RORγT gene expression is down‐regulated rather than untreated T cells. Wilcoxon matched‐pairs signed rank test was used to evaluate the statistical significant between the treated and untreated group (*n* = 13 per group). ALL, acute lymphoblastic leukaemia; Foxp3, Forkhead box protein P3; HD, healthy donor; RORγT, retinoic acid‐related orphan receptor gamma t

To determine the association between T‐cell dysfunction and LEXs in ALL patients, T‐cell transcription factors expression were analysed after 24 h co‐incubation of HD T cells with LEXs. Our data showed up‐regulated expression level of FOXP3 compared with the untreated group (1.439 ± 0.3962 versus 0.9992 ± 0.07182, *p* = 0.0068), while the expression level of RORγT was down‐regulated compared with untreated group (0.6308 ± 0.2989 versus 1.000 ± 0.05568, *p* = 0.0007) (Figure 4). These observations suggest that LEXs induce an immunosuppressive phenotype.

### 3.5 Effect of leukaemia‐ derived exosomes on cytokine production by HD T cell

**FIGURE 5:**
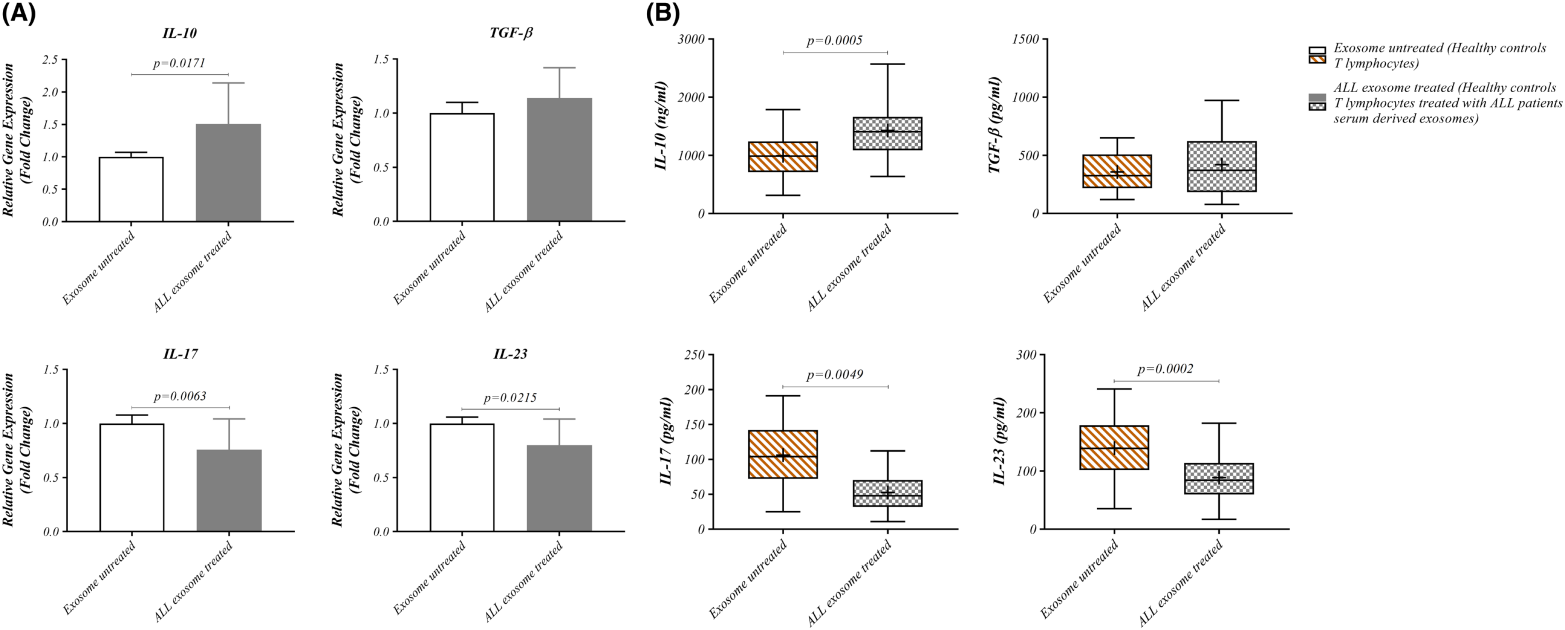
Effect of ALL cell‐derived exosomes on cytokines related to T cells. T cells from HDs co‐incubated with exosomes isolated from patients with ALL and the gene expression (A) as well as also the results of ELISA (B) of IL‐10 and TGF‐b cytokines increased in T cells treated with exosomes rather than T cells untreated with exosomes. But the gene expression (A) and also the results of ELISA (B) of IL‐17 and IL‐23 cytokines decreased in T cells treated with exosomes rather than T cells untreated with exosomes. Wilcoxon matched‐pairs signed rank test was used to evaluate the statistical significant between the treated and untreated group (*n* = 13 per group). ALL, acute lymphoblastic leukaemia; HD, healthy donor; IL, Interleukin

To evaluate that ALL patients‐derived exosomes play a role in T‐cells cytokine production, HD T cells were incubated with LEXs for 24 h. Data revealed that the expression level of IL‐10 and TGF‐B were up‐regulated in LEXs‐treated group compared with the untreated group (1.508 ± 0.6311 versus 1.000 ± 0.06843, *p* = 0.0171 and 1.140 ± 0.2803 versus 1.002 ± 0.09780, *p* > 0.05, respectively). However, the expression level of IL‐17 and IL‐23 was down‐regulated in LEXs‐treated group compared with the untreated counterparts (0.7577 ± 0.2849 versus 1.000 ± 0.07789, *p* = 0.0063 and 0.8000 ± 0.2407 versus 1.000 ± 0.05859, *p* = 0.0215, respectively) (Figure 5A).

### 3.6 Effect of leukaemia‐derived exosomes on HD T cell cytokine secretion

Furthermore, we evaluated the effects of leukaemia‐derived exosomes on T cells cytokine production. Data revealed an increase in IL‐10 and TGF‐β cytokines production in LEXs‐treated group (1430 ± 493.7 versus 993.4 ± 388.7, *p* = 0.0005 and 419.7 ± 268.5 versus 357.8 ± 162.3, *p* > 0.05, respectively). However, IL‐17 and IL‐23 cytokines were decreased after exposure to LEXs compared with the untreated group (52.54 ± 27.96 versus 106.1 ± 47.11, *p* = 0.0049 and 88.85 ± 42.47 versus 139.5 ± 54.20, *p* = 0.0002, respectively). These results suggest that exosomes from leukaemia cells may impact immune T cells by increasing immunosuppressive cytokine secretion (Figure 5B).

## 4 DISCUSSION

Exosomes have been emerged as vehicles for information transfer between cells.^44^ Although information shuttling can be the main biological function of exosomes, this connection appears to regulate molecular and genetic signs of both normal and abnormal cells. Therefore, tumour‐derived EVs have been implicated in oncogenes delivery from tumour cells to normal cells and leading to both stimulation and suppression of tumour‐specific and nonspecific immune responses.^45^, ^46^

In the present study, TEM measurements confirmed that the isolated vesicles were exosomes. Previous studies have also indicated that the vesicles obtained from leukaemia cells present biophysical characteristics of exosomes and LEXs contain known parental cell‐associated antigens^47^ and deliver their genetic information, including mRNA to recipient cells^48^ and epigenetically can reprogram target cells.^49^ Regarding the nature of the recipient cell, these EVs can be internalized.^50^

We supposed that LEXs serve an induction change in the level of mRNA expression in T lymphocytes. Thus, we co‐incubated isolated healthy human T cells with LEXs derived from sera of children with ALL. Signals induced by co‐incubated LEXs with T cells clearly showed functional results, such as up‐regulation of FOXP3 at the transcriptional level and increased IL‐10 and TGF‐B cytokines production related to Treg cells as well (Figure 4). As it has been observed in the other studies, TEXs but no other MVs or exosomes derived from normal cells, potentially increased the frequency of Treg cells and the phosphorylation of the relevant transcription factor, FOXP3.^22^ Additionally, these nanovesicles can carry TGFβ and IL‐10, the cytokines known to stimulate the conversion of conventional T‐lymphocytes into Treg cells which induce Treg expansion and enhance their functions to suppress antitumor CD8+ T cells responses to permit tumour growth as well.^35^

Because of the limited studies on Th17 cells in leukaemia, we investigated the effect of LEXs on the key transcription factor of Th17 cells, RORγT, and its related cytokines, including IL‐17 and IL‐23. RORγT and cytokines expression were lower in T lymphocyte co‐incubated with exosomes compared with the untreated group.

Although the results attributed to the Th17 cells are controversial, some studies have reported a significant decrease of Th17 cell proportion and its related cytokine, IL‐17.^51^, ^52^ In agreement with our findings, a study on CLL patients also indicated increasing in the frequency of Tregs and decreasing the frequency of Th17 cells.^53^ Decreased Th17 cells frequency, transcription factors and related cytokines were also associated with the severity of the disease, so that the number of these cells in patients with advanced leukaemia was lower than that of non‐advanced counterparts.^53^, ^54^ Additionally, it has been shown that pro‐inflammatory cytokines, including IL‐17 and IL‐23, reduce the surveillance of CD8+ T cell.^55^ In contrast, some other studies have shown that IL‐23 can stimulate T and natural killer (NK) cells. Accordingly, it exerts antitumor activity.^56^, ^57^, ^58^

This study pretends to highlight the role of LEXs to reach similar conclusions which confirm previous results. Changes in gene expression show that exosomes try to alter the immune system behaviour toward tolerance. It has long been recognized that soluble factors derived from tumour cells, such as inhibitory cytokines, have suppressive effect on the responses of immune cells which contribute in tumour progression.^59^, ^60^ Our findings are in line with previous studies and evidence shows that LEXs can promote tumour cells escape from immune system by impairing the antitumor role of T immune cells through the development of different biological proteins such as TGFβ or IL‐10 on exosomes. These results can indicate that LEXs tend to reduce Th17 cells instead of Treg cells (Figure 5A,B).

Although the crosstalk between cancer cells and immune cells is mostly accepted,^61^, ^62^, ^63^ the underlying mechanisms remain unclear until now. This study aims at investigating the immunosuppressive effect of LEXs on T cells from HDs by induction of apoptosis (Figure 3). Our study showed that LEXs promoted the apoptosis. Additionally, Ludwig et al.^39^ reported that compared with exosomes obtained from patients with no evident disease (NED), exosomes of patients with active disease (AD) mainly induced CD8+ T cells apoptosis, suppressed CD4+ T cell proliferation and increased Treg cells suppressor functions. In the present study, LEXs enhanced BAX gene expression and T cells apoptosis via caspase‐3 and ‐9 pathways. Furthermore, it has been exhibited that the co‐incubation of human T cells with LEXs mediated apoptosis in T cells. As other studies demonstrated that Fas/FasL‐driven apoptosis has been mediated by TEXs in activated CD8+ T cells.^19^, ^38^, ^64^ Moreover, a number of cancerous patients express programmed cell death 1 (PD‐1) in their peripheral blood.^65^ TEXs in sera of these patients transfer the ligand of Fas and/or PD1. Hence, the Fas/FasL or PD 1/PD L1 would be responsible for the spontaneous apoptosis observed in CD8+ T cells.^66^ In addition, another study revealed that CD4+ CD25neg T‐cells co‐incubated with TEXs converted to CD4+ CD25highFOXP3+ T cells which were completely resistant to the TEXs‐mediated apoptosis.^19^

In conclusion, exosomes in sera of patients with ALL carry immunosuppressive molecules and interfere with the role of immune cells through converting the Treg phenotype and reducing inflammatory cytokines which would be correlated with the function of exosomes not only as liquid biomarkers in diseases activity and cancer stage, but also for the level of immune suppression.

## AUTHOR CONTRIBUTIONS

**Elham Gholipour:** Investigation (equal); methodology (equal); writing – original draft (equal). **Hooman Kahroba:** Investigation (equal); methodology (equal). **Nasim Soltani:** Investigation (equal); methodology (equal). **Parisa Samadi:** Investigation (equal); methodology (equal). **Parisa Sarvarian:** Investigation (equal); methodology (equal). **Sajjad Vakili‐Samiani:** Data curation (equal). **Abbas Ali Hosein Pour Feizi:** Data curation (equal). **Mohammad Sadegh Soltani‐Zangbar:** Formal analysis (equal); software (equal). **Adel Baghersalimi:** Data curation (equal). **Bahram Darbandi:** Data curation (equal). **Aliakbar Movassaghpour:** Conceptualization (equal); methodology (equal); project administration (equal). **Mehdi Talebi:** Formal analysis (equal). **Roza Motavalli:** Investigation (equal); methodology (equal). **Amir Mehdizadeh:** Writing – review and editing (equal). **Abdolhassan Kazemi:** Writing – review and editing (equal).

## CONFLICT OF INTEREST

The authors declare no conflict of interest.

## Supporting information


Table S1

## Data Availability

The data sets used and/or analyzed during the current study are available from the corresponding author on reasonable request.
