# Supplementary material for: Paediatric pre‐B acute lymphoblastic leukaemia‐derived exosomes regulate immune function in human T cells
Source: J Cell Mol Med. 2022 Jul 13;26(16):4566–76. doi: 10.1111/jcmm.17482 (PMC9357647; doi:10.1111/jcmm.17482)
Supplement: Supplementary file 1 — Table S1 [file JCMM-26-4566-s001.docx]

**Table S1 Characteristics of the B-cell precursor ALL patients (n = 13)**

| Sample type | PB/BM | PB/BM | PB/BM | PB/BM | PB/BM | PB/BM | PB/BM | PB/BM | PB/BM | PB/BM | PB/BM | PB/BM | PB/BM |
| --- | --- | --- | --- | --- | --- | --- | --- | --- | --- | --- | --- | --- | --- |
| Age  at diagnosis (y) | 5  yrs | 7  yrs | 6  yrs | 3  yrs | 2  yrs | 4  yrs | 6  yrs | 4  yrs | 12  yrs | 11  yrs | 4  yrs | 10  yrs | 3  yrs |
| Gender  (Male/  Female) | F | F | M | M | M | F | M | M | M | M | F | M | M |
| WBC^*^  (× 10^3^ / µL) | 22/5 | 6/5 | 3/4 | 8/5 | 24/9 | 2/5 | 9/9 | 3/3 | 6/5 | 70/41 | 48/2 | 29/7 | 38/72 |
| Neutrophil^*^  ( %) | 8 | 44 | 6/7 | 0/4 | 6 | 8/1 | 24 | 7/7 | 43/1 | 20/6 | 1 | 7 | 0/4 |
| Lymphocyte^*^  ( %) | 90 | 55 | 90 | 99/5 | 72 | 91 | 66 | 90/5 | 49/1 | 64/7 | 92 | 71 | 98/4 |
| Blast^**^ (%) | 31.4 | 88 | 93 | 92.4 | 88.4 | 37 | 81 | 72 | 77/5 | 85 | 39 | 68 | 78 |
| RBC^*^  (× 10^6^ / dL) | 1/8 | 2 | 1/5 | 1/6 | 1/3 | 3 | 1/5 | 2/52 | 3/20 | 1/50 | 1/05 | 1/4 | 0/81 |
| Hb (g/dL) | 5/7 | 6/3 | 9/2 | 6/3 | 6/8 | 7/5 | 9/2 | 7 | 9/8 | 1/6 | 2 | 2/8 | 2/5 |
| Platelet^*^  (× 10^3^ / µL) | 20 | 80 | 63 | 140 | 61 | 141 | 63 | 54 | 54 | 330 | 7 | 57 | 12 |
| B cell markers^**^ :  CD19/ CD20 | 95%  /neg | 67% /2.1 | 93% /1% | 77% / 5% | 74% / 60% | 50% / 4.2% | 83% /2% | 95% /1% | 74% /2.5 | 16% / 16% | 75% / 59% | 74% / 65% | 69% / 3% |
| T cell markers^**^:  CD3/ CD4/ CD5/ CD7 | neg/ neg/ neg/ neg/ | 4.9%  /neg /4.5 /neg | neg/ neg/ neg/ neg/ | neg/ neg/ 1.6/ 2.1/ | 15%/ neg/ 14%/ neg/ | 2% / neg / 3% / | neg/ neg/ neg/ neg/ | neg/ neg/ neg/ neg/ | 5 %  /5.5 /neg /neg | 48% / neg / 22% / neg | 13%/ neg/ 16%/ neg/ | 13%/ neg/ 15%/ neg/ | 5.9%  /neg /3.5 /neg |
| Myeloid markers^**^:  CD13/ CD33 | neg/ neg/ | 34% /5% | 8% / neg | 4.5% / 0.9% | 5.1% / 2% | 1.1% / 8.5% | 9% / neg | 7% / 1% | 35% /4% | neg/ neg/ | 5.9% / 3% | 6.2% / 1% | 9% / neg |
| Non lineage markers^**^:  CD10/ HLA-DR/  CD45 | 79% / 83% /  83% | 84%/80% /74% | 94% / 85% / 70% | 94% / 55% / 18.4% | 72% / 82% / 39% | 50% / 63% / 75% | 84% / 74% / 70% | 95% / 85% / 73% | 87%/79% /75% | 1%/ 16% / 85% | 82% / 72% / 40% | 72% / 85% / 49% | 94% / 83% / 71% |
| ^*^ Detected by vein blood specimen.  ^* *^Detected by bone marrow specimen. | | | | | | | | | | | | | |
